# Neonatal pulmonary vascular remodeling induced by increased blood flow is associated with an antiviral-like immune signature

**DOI:** 10.3389/fimmu.2026.1780303

**Published:** 2026-03-04

**Authors:** Sixie Zheng, Hao Li, Siqi She, Yiting Xue, Debao Li, Jiapei Wang, Jing Wang, Yuqing Hu, Lincai Ye

**Affiliations:** 1 Department of Thoracic and Cardiovascular Surgery, Shanghai Children’s Medical Center, Shanghai Jiao Tong University School of Medicine, Shanghai, China; 2 Department of Pediatric Surgery, Children’s Hospital of Fudan University, National Children’s Medical Center, Shanghai, China; 3 Department of Pediatric Critical Care Medicine, The Affiliated Women and Children’s Hospital of Ningbo University, Ningbo, Zhejiang, China; 4 Department of Infectious Diseases, Shanghai Children’s Medical Center, Shanghai Jiao Tong University School of Medicine, Shanghai, China; 5 Department of Cardiology, Shanghai Children’s Medical Center, Shanghai Jiao Tong University School of Medicine, Shanghai, China; 6 Shanghai Institute For Pediatric Congenital Heart Disease, Shanghai Children’s Medical Center, Shanghai Jiao Tong University School of Medicine, Shanghai, China

**Keywords:** increased pulmonary blood flow, left-to-right shunt, lung, neonatal animal models, pulmonary vascular remodeling

## Abstract

**Background:**

Approximately 70% of pediatric pulmonary arterial hypertension (PAH) is associated with congenital heart disease causing increased pulmonary blood flow (IPF). The developing neonatal lung is highly susceptible to hemodynamic stress, yet the direct causal link and mechanisms of neonatal IPF-induced pulmonary vascular remodeling remain poorly understood due to the lack of suitable animal models that recapitulate this critical developmental window.

**Methods:**

We established a novel neonatal mouse model of IPF by performing an aortocaval fistula (ACF) on postnatal day 7(P7). Pulmonary hemodynamics were assessed by ultrasound at P30. Vascular remodeling was evaluated through histology (H&E, α-SMA immunofluorescence) and molecular analysis of phenotypic markers (Spp1, Myh11). Transcriptomic profiling (RNA-seq) and pathway enrichment analysis were employed to uncover underlying mechanisms, with flow cytometry and immunosuppression (Cyclosporin A) and type I interferon receptor blocker (MAR1-5A3) used for functional validation.

**Results:**

The neonatal ACF model successfully induced a left-to-right shunt, resulting in significant IPF and right ventricular volume overload. IPF mice exhibited pronounced pulmonary small vessel remodeling, evidenced by increased α-SMA intensity, elevated synthetic-phenotype marker (Spp1) expression, and decreased contractile-phenotype marker (Myh11). Transcriptomic analysis revealed a dominant immune signature, with the most enriched pathways being antiviral and interferon-response related (response to virus and IL-17 signaling). This was corroborated by a significant increase in pulmonary CD4+ and CD8+ T cells. Crucially, immunosuppressive treatment and type I interferon receptor blocker attenuated vascular remodeling.

**Conclusions:**

We provide the direct experimental evidence that neonatal IPF alone is sufficient to drive pulmonary small vessel remodeling. The process is fundamentally mediated by an activated immune response characterized by an antiviral-like signature, a mechanism distinct from those reported in classic adult PAH models. This novel model offers a critical platform for investigating the developmental-specific pathogenesis of pediatric PAH and bridging the translational “valley of death.”

## 1 Introduction

Pulmonary arterial hypertension (PAH) in children represents a severe clinical challenge with distinct pathophysiology from adult disease (1–3). Notably, approximately 70% of pediatric PAH cases are associated with increased pulmonary blood flow (IPF), primarily due to congenital cardiac left-to-right shunts (4–6). This highlights IPF as a predominant causative factor in this population. The developing pediatric lung, particularly during the rapid postnatal alveolar and vascular maturation phase (approximately postnatal days 0–30 in rodents), exhibits unique vulnerability (7–10). However, a critical gap exists in preclinical research: there is a notable lack of animal models that faithfully recapitulate PAH development during this specific developmental window (11–13). Traditional models induced by chronic hypoxia or agents like monocrotaline in adult animals fail to capture the complex interplay of hemodynamic stress and ongoing lung development (11–13).

This translational disconnect is further evidenced by the so-called “valley of death” in PAH therapeutics (11, 14). Numerous compounds demonstrating remarkable efficacy in standard adult animal models have subsequently failed in human clinical trials (11, 14), underscoring the inadequacy of existing models for predicting clinical success, especially for pediatric PAH. To address this gap, we previously established a neonatal mouse model of IPF by creating an aortocaval fistula (ACF) to induce a left-to-right shunt (15–18). This model successfully recapitulated key clinical features, including right ventricular volume overload and IPF, leading to impaired alveolar development (15–18).

Building upon this foundation, the present study aims to investigate a pivotal, yet experimentally unconfirmed, clinical sequela: whether neonatal IPF directly instigates pulmonary small vessel remodeling, the fundamental pathological lesion of PAH. Furthermore, we preliminarily explore the underlying molecular mechanisms. By establishing a direct causal link between developmental-stage-specific IPF and vascular pathology, this work seeks to provide a novel and more clinically relevant platform. This model holds significant potential for elucidating the mechanisms of pediatric PAH progression and for bridging the “valley of death” by facilitating the testing of therapeutic strategies in a context that mirrors the human disease condition more accurately.

## 2 Materials and methods

### 2.1 Data availability and ethics statement

The RNA-seq datasets generated and analyzed during the current study are available in the GEO repository under the accession number GSE291221. All other relevant data supporting the findings are available from the corresponding author upon reasonable request. All animal experimental procedures were reviewed and approved by the Shanghai Children’s Medical Center Institutional Animal Care and Use Committee (IACUC Approval No. SCMC-LAWEC-2024-1127). All methods were performed in accordance with the relevant guidelines and regulations (e.g., ARRIVE guidelines).

### 2.2 Study design

**Figure 1:**
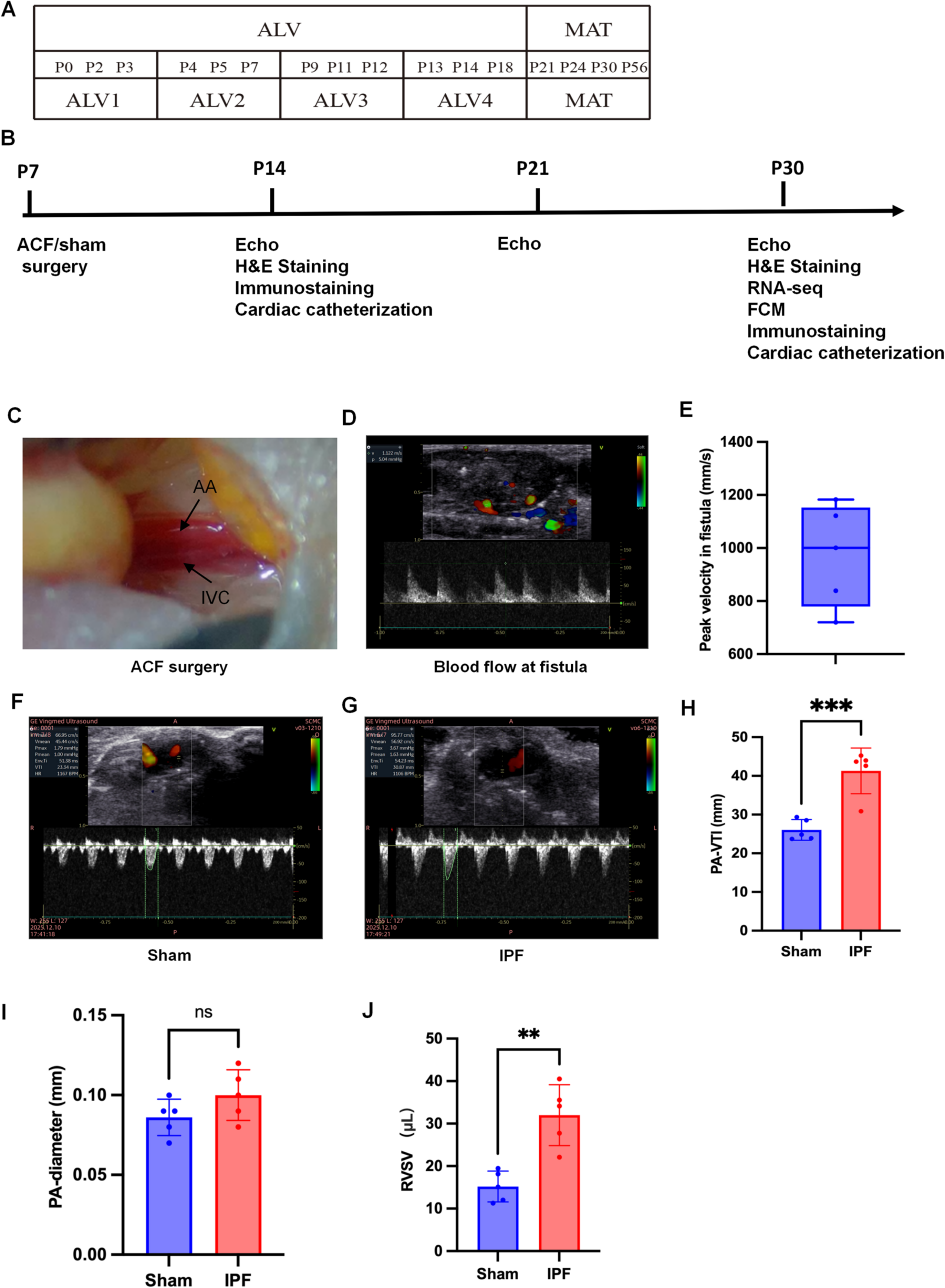
Establishment and validation of the neonatal increased pulmonary flow (IPF) model. **(A)** Schematic timeline of mouse postnatal lung development. **(B)** The experimental design. ACF surgery was performed at postnatal day 7 (P7, alveolar stage), with analyses at P30 (maturation stage). **(C)** Representative bright-field image of the site for ACF surgery. **(D, E)** Abdominal ultrasound imaging confirming turbulent shunt flow and its velocity (550 mm/s). **(F–H)** Echocardiography showing a significant increase in the pulmonary artery velocity time integral (PA-VTI). **(I)** Quantification of pulmonary artery diameter, showing no significant change. **(J)** Quantification of right ventricular stroke volume (RVSV), demonstrating a significant increase. Data are mean ± SD; **p < 0.01, ***p < 0.001 vs. Sham group (unpaired t-test). n = 5 per group.

This study was designed to investigate the impact of neonatal IPF on pulmonary small vessel remodeling and its underlying immune-related mechanisms (**Figures 1A, B**). Neonatal C57BL/6J mice were randomly assigned to either the IPF model group (subjected to aortocaval fistula surgery, ACF) or the sham-operated control group. The primary endpoint was the assessment of pulmonary vascular remodeling via histological and molecular analyses at postnatal day 30 (P30). Secondary endpoints included hemodynamic validation by ultrasound, transcriptomic profiling, and immunophenotyping. An additional interventional arm involved treating a subset of IPF mice with the immunosuppressant Cyclosporin A (CsA, 20 mg/kg/3 day, starting at P14 and continued until P30) or type I IFN receptor blocker MAR1-5A3 (GTX14637, Beyotime, Shanghai, 12.5mg/kg/2 day) to evaluate the functional role of the immune response.

The primary discovery cohort consisted of sham-operated and ACF (IPF) mice used for hemodynamic, histological, and transcriptomic analyses. Interventional studies (CsA and type I IFN receptor blockade) were performed in independent experimental cohorts separate from the primary discovery cohort. In each interventional cohort, IPF mice receiving drug treatment and corresponding IPF control mice receiving vehicle were generated within the same surgical and experimental batch to minimize batch effects. Sham-operated animals from the same time period were used as reference controls when appropriate.

Due to the limited tissue volume of neonatal mouse lungs, cohort usage and tissue allocation were predefined before experiments. Separate animal cohorts were used for major assay categories to ensure adequate sample quality and avoid cross-assay interference. Specifically, whole lung tissue from designated animals was used for RNA sequencing, perfused and fixed lungs were used for histological and immunofluorescence analyses, and freshly dissociated lungs were used for flow cytometry.

qRT-PCR validation was performed using RNA derived either from the RNA-seq cohort or from an independent validation cohort processed in parallel. Each animal was assigned to a primary assay category at the time of sacrifice and was not reused across incompatible downstream assays.

### 2.3 Animals and ACF surgery

Timed-pregnant C57BL/6J mice were raised in our laboratory. Pups of both sexes were used on postnatal day 7 (P7) for surgery. Animals were randomly assigned to sham or ACF groups at the time of surgery, with sex-balanced allocation performed when feasible within each litter. No animals were excluded based on sex. Perioperative survival was recorded prospectively. Deaths occurring within 24 h after surgery were defined as surgery-related mortality. Early (24–72 h) and late (>72 h to endpoint) mortality were also recorded. Survival and baseline characteristics, including sex distribution and body weight at surgery, are summarized in **Supplementary Table 1**.

Mice were anesthetized by isoflurane inhalation (2-3% for induction, 1-2% for maintenance) and placed on a heating pad. Under a stereomicroscope, a midline abdominal incision was made. The abdominal aorta and inferior vena cava (IVC) were carefully isolated. An 9–0 needle (0.07 mm diameter) was used to create a puncture between the adjacent vessels, followed by a 11–0 nylon suture (Ethicon) to establish a permanent fistula. Patency was confirmed by visual observation of pulsatile, turbulent flow. For sham operations, vessels were isolated but not punctured. The abdominal wall and skin were closed in layers. Pups were warmed until recovery and returned to the dam. Analgesia (buprenorphine, 0.05 mg/kg) was administered postoperatively.

### 2.4 Abdominal ultrasound and echocardiography

At P30, mice were anesthetized with 1.5% isoflurane. Abdominal ultrasound was performed using a Vevo 3100 high-resolution imaging system (Fujifilm VisualSonics) with an MX550D transducer to visualize the ACF fistula and measure shunt flow velocity. Transthoracic echocardiography was then conducted. Pulmonary artery outflow was assessed in a parasternal short-axis view to measure the pulmonary artery velocity time integral (PA-VTI) and diameter. Right ventricular (RV) dimensions and function were analyzed from an apical four-chamber view. Right ventricular stroke volume (RVSV) was calculated as: RVSV [mL] =1/4 × πD^2^× PA-VTI. All measurements were averaged over three consecutive cardiac cycles by an investigator blinded to group allocation.

### 2.5 Cardiac catheterization

Hemodynamic measurements were obtained by cardiac catheterization using an open-chest approach under general anesthesia. After tracheal intubation and mechanical ventilation, a left thoracotomy was performed to expose the heart. Millar catheter transducer (ADInstruments, SPR-671NR) was directly inserted into the right ventricle under visual guidance and further advanced into the pulmonary artery for continuous pressure recordings. Right ventricular systolic pressure (RVSP) and pulmonary arterial pressure were recorded and analyzed using PowerLab system (ADInstruments). Hemodynamic parameters were derived from stable waveform segments.

### 2.5 Hematoxylin and eosin staining

After hemodynamic assessment, lungs were perfused with PBS via the right ventricle, followed by inflation and fixation with 4% paraformaldehyde (PFA) at a constant pressure of 25 cm H_2_O. Tissues were paraffin-embedded and sectioned at 5 μm thickness. Sections were deparaffinized, rehydrated, and stained with H&E using standard protocols.

### 2.6 Immunofluorescence

Paraffin sections underwent antigen retrieval in citrate buffer. After blocking with 5% normal donkey serum, sections were incubated overnight at 4 °C with primary antibodies: rabbit anti-α-Smooth Muscle Actin (α-SMA, 1:400, Abcam ab5694). After washing, appropriate fluorescently-labeled secondary antibodies (Alexa Fluor 594, 1:500, Invitrogen) were applied. Nuclei were counterstained with DAPI. Fluorescence images were acquired using a confocal microscope (Zeiss LSM 880). The integrated fluorescence density of α-SMA+ vessels in consistent fields of view was quantified using ImageJ.

### 2.7 RNA sequencing and analysis

Total RNA was extracted from snap-frozen whole lung tissue (n=4–5 per group) using TRIzol reagent. RNA integrity was verified (RIN > 8.0) by Bioanalyzer. Libraries were prepared with the KAPA mRNA HyperPrep Kit and sequenced on an Illumina NovaSeq 6000 platform (150 bp paired-end). Raw reads were quality-trimmed using Fastp and aligned to the mouse reference genome (GRCm39) using HISAT2. Gene expression quantification was performed with StringTie. Differential expression analysis (IPF vs. Sham) was conducted using DESeq2 with thresholds of |log2FoldChange| > 1 and adjusted p-value (q-value) < 0.05. Gene Ontology (GO) and Kyoto Encyclopedia of Genes and Genomes (KEGG) pathway enrichment analyses were performed using the clusterProfiler R package.

### 2.8 qRT-PCR

qRT-PCR was performed to validate key differentially expressed genes identified by RNA-seq. Total RNA (1 µg) was reverse transcribed into cDNA using the PrimeScript RT reagent Kit (Takara). Quantitative PCR was carried out on a LightCycler 96 System (Roche) using SYBR Green Premix Pro Taq HS (Accurate Biology). Gene expression was normalized to the housekeeping gene Gapdh and calculated using the 2^(-ΔΔCt) method. Primer sequences for target genes are listed in **Supplementary Table 2**.

### 2.9 Flow cytometry

Single-cell suspensions were prepared from perfused lung tissue by mechanical dissociation and enzymatic digestion (Collagenase IV/DNase I). Red blood cells were lysed. Cells were blocked with anti-CD16/32 antibody and stained with fluorochrome-conjugated antibodies for surface markers: BV605 anti-CD45 (30-F11), PE-Cy7 anti-CD4 (GK1.5), and APC anti-CD8a (53-6.7). Corresponding isotype controls were used. Cells were analyzed on a Beckman CYTOFLEX flow cytometer. Data were processed using FlowJo software (v10.8.1), gating on live, singlet, CD45+ leukocytes.

### 2.10 Statistical analysis

All data are presented as mean ± standard error of the mean (SEM). Statistical analyses were performed using GraphPad Prism (v9.0). Normality was assessed using the Shapiro-Wilk test. For comparisons between two groups (Sham vs. IPF), an unpaired, two-tailed Student’s t-test (parametric) or Mann-Whitney U test (non-parametric) was used. For comparisons involving multiple groups (e.g., Sham, IPF, IPF+CsA), one-way ANOVA with Tukey’s *post-hoc* test or Kruskal-Wallis with Dunn’s test was applied accordingly. A p-value < 0.05 was considered statistically significant.

## 3 Results

### 3.1 Establishment and validation of the neonatal IPF model

As depicted in **Figures 1A**, the neonatal mouse lung undergoes rapid postnatal development, progressing through the alveolar stage (ALV stage, postnatal days 0-18) followed by the lung maturation stage (M stage, days 21-56) (7–10). To create a left-to-right shunt, we performed an aortocaval fistula (ACF) surgery on postnatal day 7 (ALV2 stage). Subsequent analyses, including echocardiography, RNA-seq, and flow cytometry, were conducted on postnatal day 30 (M stage) (**Figure 1B**). Following ACF surgery (**Figure 1C**), pulsatile blood flow was clearly visible at the fistula site with a velocity of 550 mm/s (**Figures 1D, E**). Echocardiographic assessment revealed a significant increase in the velocity time integral of the pulmonary artery (**Figures 1F–H**). While the diameter of the pulmonary artery showed no significant change (**Figure 1I**), the right ventricular stroke volume (RVSV) was markedly increased (**Figure 1J**). These results indicate the successful induction of a left-to-right shunt leading to increased pulmonary blood flow during the neonatal period, consistent with clinical investigation (5, 6). Serial abdominal ultrasound confirmed shunt persistence from P7 to P30, demonstrating the model’s hemodynamic stability (**Supplementary Figures 1A, B**). Baseline characteristics, including litter size, sex distribution, and perioperative survival, showed no significant intergroup differences at surgery (**Supplementary Table 1**), supporting balanced allocation and minimizing selection bias.

### 3.2 Transcriptomic analysis confirms the robustness of the IPF model

**Figure 2:**
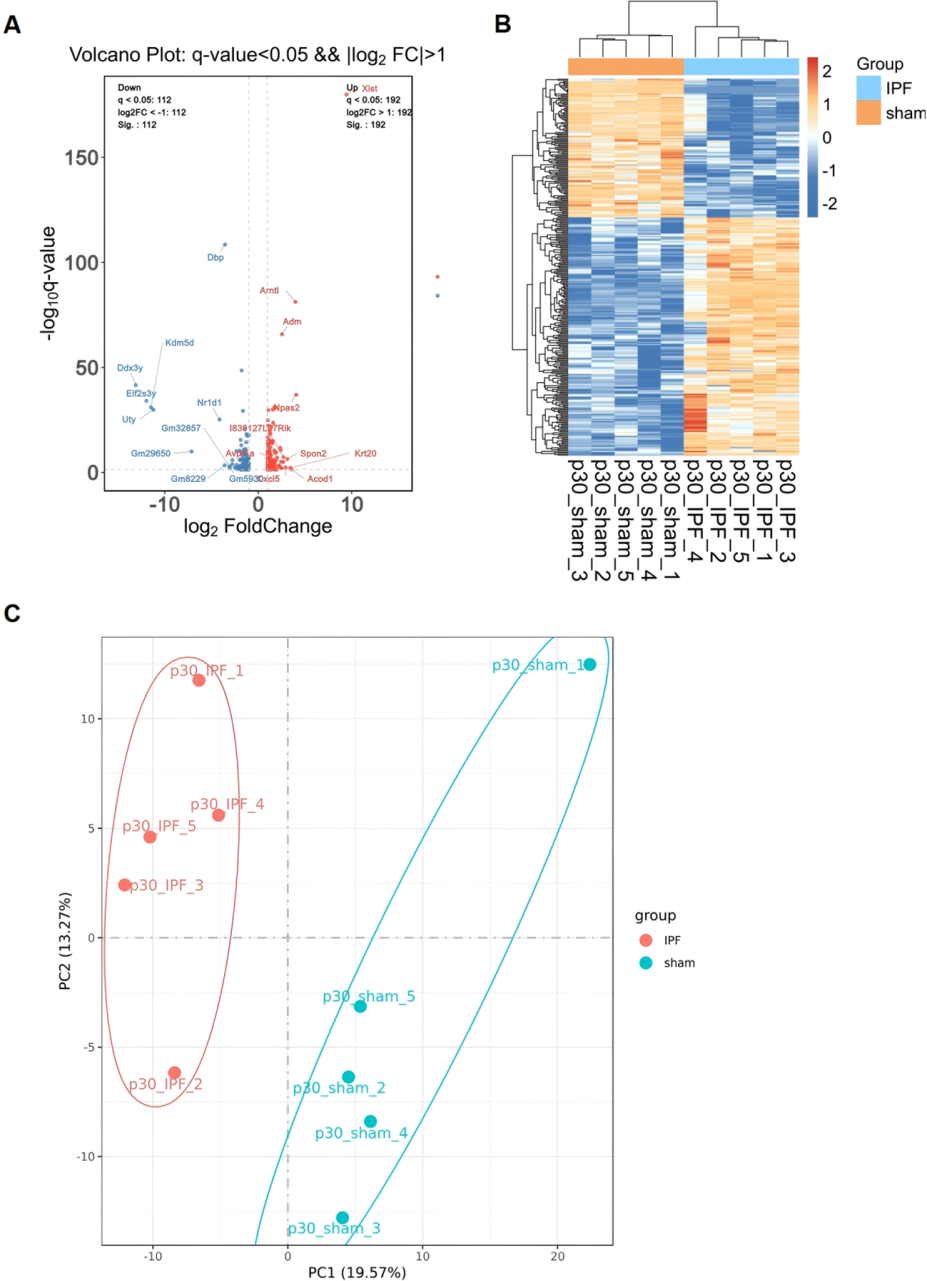
Transcriptomic profiling confirms a robust IPF-induced phenotype. **(A)** Volcano plot of differentially expressed genes (DEGs) in lung tissues from IPF vs. Sham mice (q-value < 0.05 and |log2FC| > 1). Red and blue dots represent up- and down-regulated genes, respectively. **(B)** Heatmap of hierarchical clustering analysis of the DEGs, showing clear separation between IPF and Sham groups. **(C)** Principal component analysis (PCA) plot demonstrating distinct transcriptomic profiles between the two groups.

We next performed RNA-seq analysis on the model. IPF induction resulted in 304 differentially expressed genes (DEGs) (q-value < 0.05 && |log2FC| > 1), among which 112 were upregulated and 192 were downregulated (**Figure 2A**). Cluster analysis of these DEGs demonstrated clear separation between groups and high similarity within groups (**Figure 2B**), suggesting a highly consistent pulmonary remodeling phenotype induced by IPF. This finding was further corroborated by PCA analysis, which showed distinct separation between the IPF and sham groups along principal components (**Figure 2C**).

### 3.3 Neonatal IPF significantly induces pulmonary small vessel remodeling

**Figure 3:**
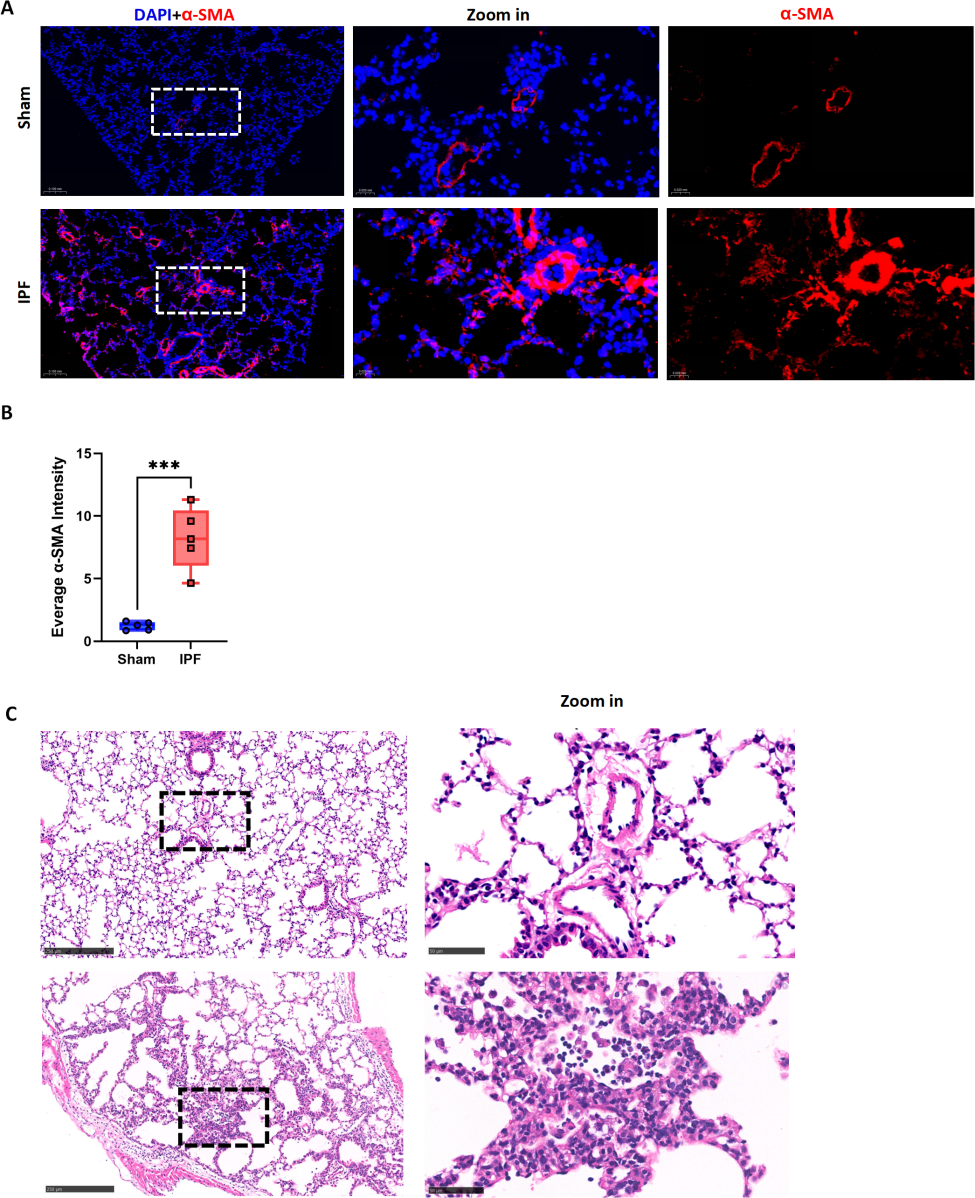
Neonatal IPF induces significant pulmonary small vessel remodeling. **(A)** Representative immunofluorescence images of lung sections stained for α-SMA (red) Nuclei are counterstained with DAPI (blue). **(B)** Quantification of α-SMA integrated fluorescence density, showing a significant increase in the IPF group. **(C)** Representative H&E staining of lung sections. Data are mean ± SD; ***p < 0.001 vs. Sham group (unpaired t-test). n = 5 per group.

**Figure 4:**
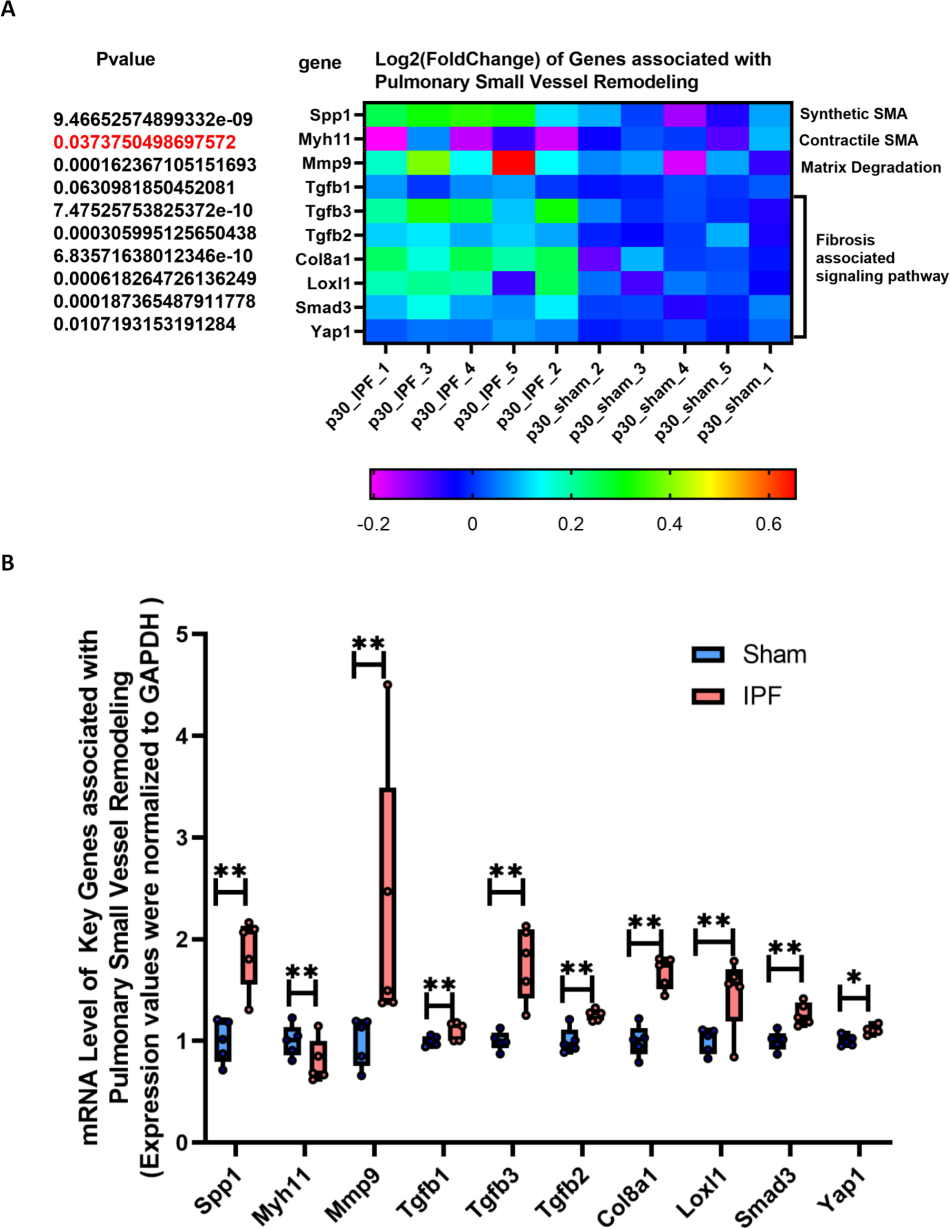
Molecular signatures of vascular remodeling and immune activation. **(A)** Heatmap displaying the expression levels of key genes involved in vascular remodeling (Spp1, Myh11, Mmp9) and signaling pathways (*Tgfb1-3, Smad3, Yap1*) from RNA-seq data. **(B)** qRT-PCR validation of the genes shown in **(A)**, confirming the RNA-seq results. Gene expression is normalized to Gapdh and presented relative to the Sham group. Data are mean ± SD; *p < 0.05, **p < 0.01 vs. Sham group (unpaired t-test). n = 5 per group.

Although clinical observation identifies IPF as a prerequisite for pediatric pulmonary hypertension (5, 6), the lack of a neonatal IPF animal model has left the direct causal relationship unconfirmed. As shown in **Figures 3A, B**, immunofluorescence staining revealed that neonatal IPF induced diffuse pulmonary small vessel remodeling, evidenced by a significant increase in the fluorescence density of pulmonary vessels (α-SMA-positive) (**Figure 3B**). This observation was further confirmed by H&E staining (**Figure 3C**). Transcriptomic analysis (**Figure 4A**) showed a significant upregulation of Spp1 (a marker gene for synthetic-phenotype SMCs) and Mmp9 (a key metalloproteinase mediating extracellular matrix remodeling in pulmonary hypertension), alongside a significant downregulation of Myh11 (a marker gene for contractile-phenotype SMCs). Furthermore, the expression of key signaling molecules mediating vascular remodeling, such as those in the TGF-β1–3 and SMAD3/YAP1 pathways, was significantly elevated. qRT-PCR validation confirmed these RNA-seq findings with high consistency (**Figure 4B**). Collectively, these results indicate that neonatal IPF contributes to the development of pulmonary small vessel remodeling.

To directly address whether pulmonary vascular remodeling represents an early and progressive consequence of neonatal IPF rather than a late adaptive phenomenon, we further performed additional time-course and hemodynamic analyses. The results showed that from postnatal day 14 (P14) to P30, both α-SMA and HE staining revealed a progressive thickening of the medial layer of pulmonary small vessels. In parallel, right ventricular systolic pressure (RVSP) measured by cardiac catheterization also showed a gradual increase (**Supplementary Figures 2A–D**).

### 3.4 Mechanistic insights into neonatal IPF-induced remodeling

**Figure 5:**
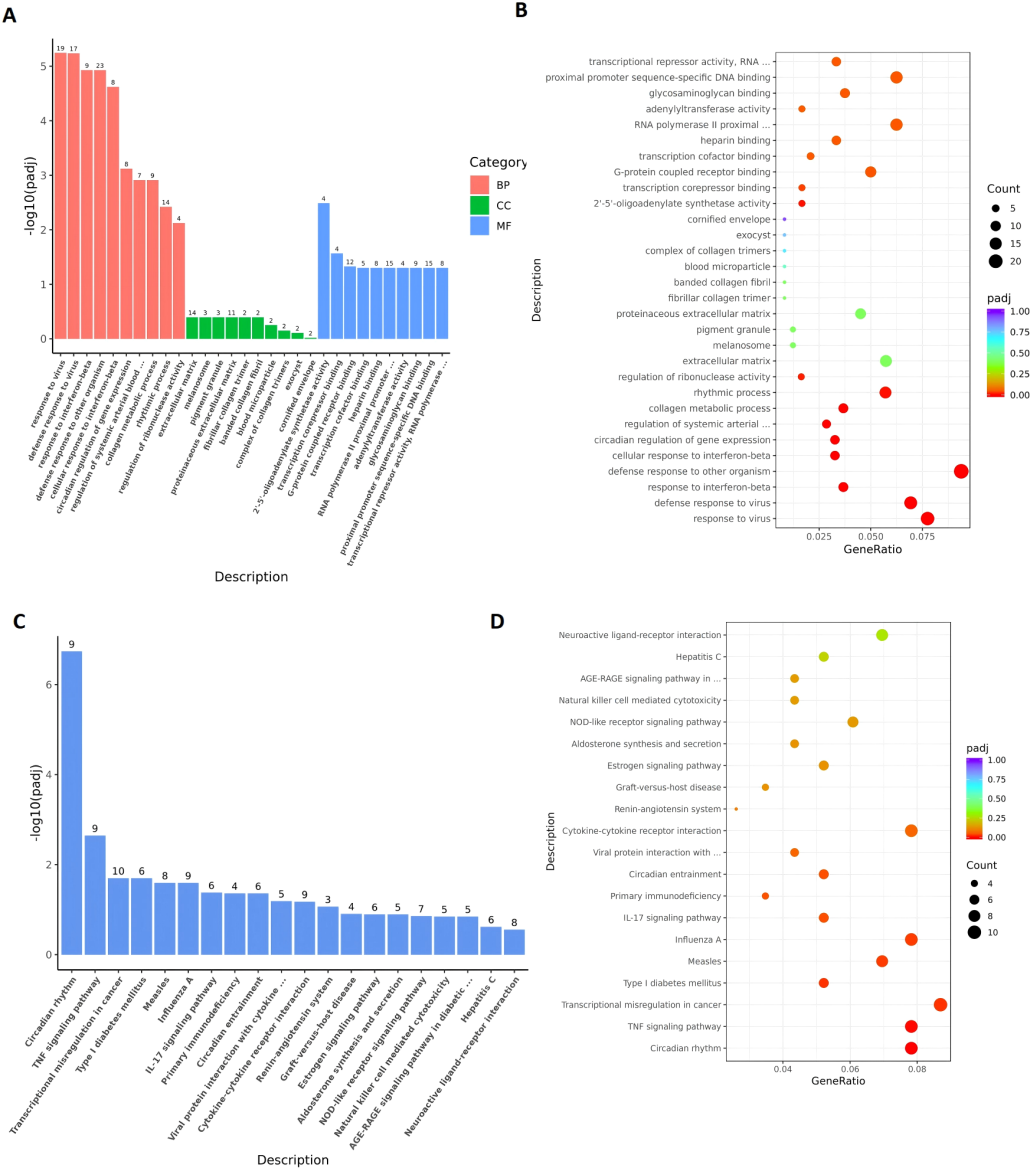
Enrichment analyses reveal dominant immune-related pathways. **(A, B)** Top 30 enriched Gene Ontology (GO) terms for all DEGs. **(C, D)** Top 20 enriched KEGG pathways for all DEGs. The enrichment score is presented as -log10(padj).

To investigate the mechanisms underlying neonatal IPF-induced remodeling, we performed enrichment analysis on the DEGs. GO enrichment analysis (**Figures 5A, B**; **Supporting Data 1**) revealed that among the top ten enriched Biological Process (BP) terms, five were related to immune response: “response to virus,” “defense response to virus,” “response to interferon (IFN)-beta,” “defense response to other organism,” and “cellular response to IFN-beta.” Within the top thirty enriched terms, eight were associated with vascular remodeling, including “regulation of systemic arterial blood pressure mediated by a chemical signal,” “collagen metabolic process,” and terms related to extracellular matrix and collagen structure. Pathway enrichment analysis (**Figures 5C, D**) indicated that 10 out of the top twenty enriched pathways were immune-related, such as the “TNF signaling pathway,” “IL-17 signaling pathway,” “Cytokine-cytokine receptor interaction,” and “Natural killer cell mediated cytotoxicity.” Enrichment analysis focusing solely on upregulated DEGs showed an even more pronounced association with immune responses (**Supplementary Figures 3A–D** and **Supporting Data 2**). These findings suggest that immune activation may represent a key mechanism in IPF-induced pulmonary vascular remodeling.

### 3.5 Immune activation is associated with vascular remodeling in IPF

**Figure 6:**
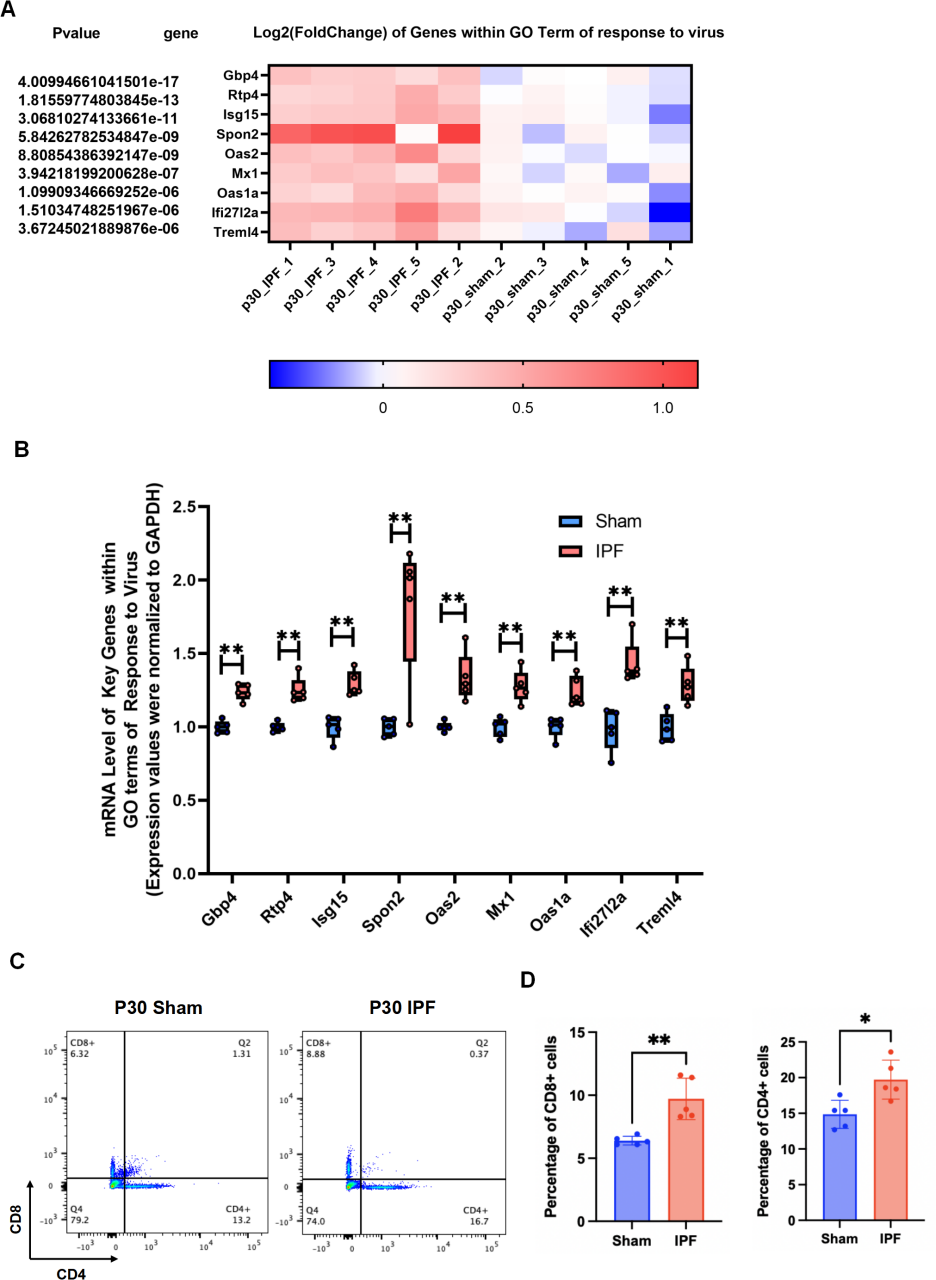
IPF activates a virus-response signature and T cell infiltration. **(A)** Heatmap displaying the expression levels of key genes involved in virus-response from RNA-seq data. **(B)** Relative mRNA expression of nine genes from the top-ranked GO term “response to virus,” as measured by qRT-PCR. **(C)** Representative flow cytometry plots showing CD4+ and CD8+ T cell populations. **(D)** Quantification of CD4+ and CD8+ T cell percentages in the lungs. Data are mean ± SD; *p < 0.05, **p < 0.01, vs. Sham group (unpaired t-test). n = 5 per group.

To experimentally validate the transcriptomic signature indicative of an antiviral-like response, we first examined the expression of key differentially expressed genes (DEGs) within the top-ranked GO term “response to virus.” qRT-PCR analysis confirmed the significant upregulation of all nine tested DEGs in IPF lungs compared to controls (**Figures 6A, B**). This molecular signature was further corroborated at the cellular level by flow cytometry (**Figures 6C, D**). While the absolute increase in T cell frequency was modest (~2–3 percentage points), the change was statistically significant and occurred in the context of a globally low inflammatory milieu, suggesting a specific immune activation in response to increased flow.

**Figure 7:**
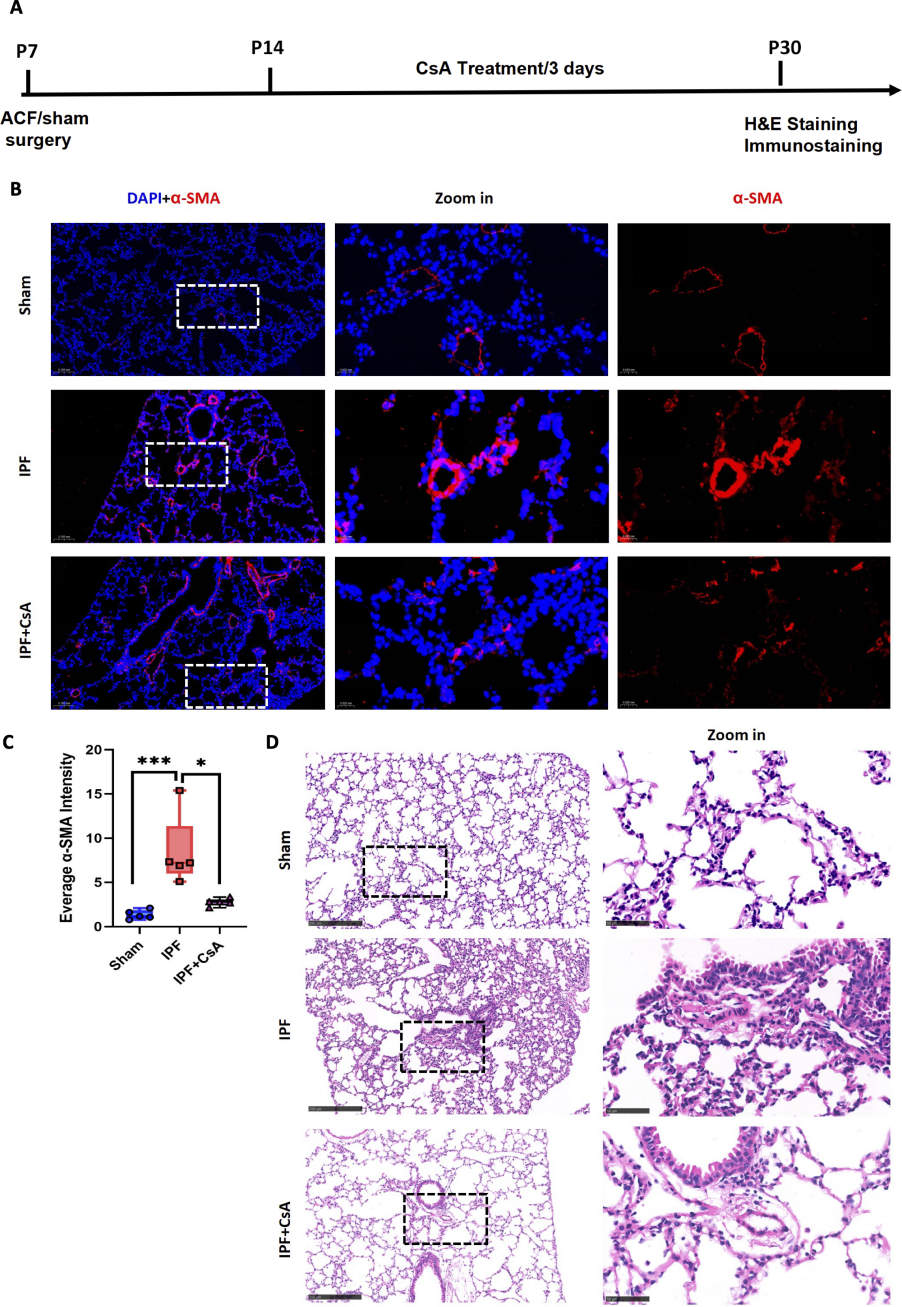
Immunosuppression attenuates IPF-induced vascular remodeling. **(A)** CsA intervention schedule. **(B)** Representative immunofluorescence images of lung sections (α-SMA in red). **(C)** corresponding quantification of α-SMA fluorescence density from Sham, IPF, and IPF+CsA groups. **(D)** Representative H&E staining of lung sections. Data are mean ± SD; *p < 0.05,* **p < 0.001 (one-way ANOVA with Tukey’s *post-hoc* test). n = 5 per group.

We next investigated whether broad immunosuppression could mitigate the resultant pathology. Treatment of IPF model mice with the immunosuppressant CsA significantly attenuated IPF-induced remodeling of the pulmonary small vasculature (**Figures 7A–D**). To specifically probe the contribution of the antiviral-like response, we administered the type I interferon receptor blocker MAR1-5A3. This targeted intervention similarly led to a significant reduction in pulmonary vascular remodeling (**Supplementary Figures 4A–C**).

Collectively, these data demonstrate that the activation of an antiviral-like immune response is associated with pulmonary small vessel remodeling in neonatal IPF and is functionally involved in the remodeling phenotype, as suggested by attenuation with immunomodulatory interventions.

## 4 Discussion

Building upon our previous work, which centered on impaired alveolar development, cardiopulmonary hemodynamic adaptation, and right ventricular immune activation in postnatal volume overload (16–18), the present study elucidates novel mechanistic layers underlying pulmonary vascular remodeling. These include: direct histological and molecular evidence of small pulmonary vessel remodeling and smooth muscle phenotypic switching (characterized by upregulated Spp1 and downregulated Myh11), pathway-level identification of a dominant type I IFN/antiviral-like immune signature, and the functional attenuation of remodeling through immunosuppression (Cyclosporine A) and IFN-receptor blockade (MAR1-5A3). Our findings support a strong association between neonatal increased pulmonary flow and pulmonary vascular remodeling and further suggest that a type I interferon/antiviral-like immune program is functionally linked to this remodeling phenotype. This necessitates a critical comparison with the immunological landscape described in classical pulmonary arterial hypertension (PAH) models, revealing a potentially fundamental divergence with significant translational implications.

In canonical adult PAH models—such as those induced by chronic hypoxia or monocrotaline—the immune response is predominantly characterized as a form of sterile inflammation (19–21). The primary triggers are hypoxic stress or direct endothelial toxin injury, leading to the release of damage-associated molecular patterns (DAMPs) (22, 23). This typically results in the recruitment and activation of innate immune cells like macrophages and neutrophils, with a cytokine profile centered on IL-1β, IL-6, and TNF-α. While the adaptive immune system, particularly T cells, may be involved, it often operates within this broader inflammatory context (24, 25).

In stark contrast, our neonatal IPF model reveals an immune signature overwhelmingly skewed toward antiviral defense and type I IFN responses. GO and KEGG analyses highlight the most significantly enriched pathways as “response to virus,” “defense response to virus,” and “response to IFN-beta.” This pattern likely reflects a conserved type I interferon–dominant stress/immune program that can be triggered by non-infectious stimuli (e.g., mechanical stress or endothelial injury), rather than evidence of a literal viral-mimicking process. This immune activation is not merely transcriptional; it is functionally substantiated by a significant influx of CD4+ and CD8+ T cells and the demonstrable attenuation of vascular remodeling following treatment with the immunosuppressant CsA and IFN-receptor blocker MAR1-5A3.

This dichotomy—sterile injury-induced inflammation versus flow-induced antiviral mimicry—may lie at the heart of the translational “valley of death” in PAH therapeutics. Pharmacological agents developed and validated in adult models effectively target pathways relevant to hypoxia or generalized inflammation but may completely overlook the dominant IFN-driven and cytotoxic T-cell axes activated in early-life, flow-induced PAH (26–28). By recapitulating this unique immunophenotype, Our model provides a crucial explanatory platform for these clinical discrepancies. It posits that the immunological basis of pediatric PAH associated with congenital left-to-right shunts is distinct, potentially necessitating therapies that modulate specific antiviral-like pathways or T-cell responses rather than broad-spectrum anti-inflammatory agents.

In summary, the translational relevance of this model is underscored by three key aspects:(1) Developmental Specificity: It captures the critical postnatal window of lung and vascular maturation, a facet absent in adult models. (2) Hemodynamic Etiology: Remodeling is driven by chronic flow overload from a left-to-right shunt, rather than hypoxia or toxin exposure, closely mirroring the pathogenesis of congenital heart disease-associated pediatric PAH. (3) Distinct Immune Phenotype: The identified immune program fundamentally differs from the sterile inflammatory profiles of adult PAH models, suggesting that therapeutic targets derived from adult systems may lack efficacy in the pediatric setting.

In conclusion, beyond establishing the causative role of neonatal IPF in initiating vascular remodeling, our work emphasizes that the nature of the ensuing immune response is critically dependent on both the model etiology and the developmental stage. The neonatal IPF model unveils a previously underappreciated immunopathogenic pathway, offering a novel and clinically relevant framework for developing targeted interventions aimed at the specific immune dysregulation occurring in the developing lungs of children with left-to-right shunts. Future studies employing single-cell and spatial transcriptomic analyses will be essential to resolve the precise cellular origins and spatial interactions underpinning this unique immune program.

Several limitations of this study should be acknowledged. First, transcriptomic and RT–qPCR analyses identify pathway associations and directionally consistent expression changes but do not, by themselves, establish a singular causal mechanism. Second, bulk RNA sequencing lacks cell-type resolution, and future studies using single-cell or spatial transcriptomic approaches will be required to define cellular sources and intercellular signaling interactions more precisely. Third, vascular structure was defined using established morphological and smooth muscle marker criteria, but additional endothelial–smooth muscle co-staining would further refine structural specificity. Finally, although pharmacologic and receptor-blocking interventions provide functional support for immune pathway involvement, more cell-type–specific genetic approaches will be required to delineate precise causal pathways.

## Data Availability

The datasets presented in this study can be found in online repositories. The names of the repository/repositories and accession number(s) can be found below: GSE291221 (GEO).

## Supplementary material

Supplementary Figure 1 Shunt patency and stability revealed by abdominal ultrasound imaging. **(A)** Representative of abdominal ultrasound imaging at P14 and P21. **(B)** Quantification of peak velocity at the fistula.

Supplementary Figure 2 Time-course analyses of pulmonary vessels remodeling and pulmonary vascular resistance. **(A)** Representative of pulmonary vessels imaging at P14 and P30. **(B)** Quantification of small pulmonary vessels median thickness(%). **(C)** Representative of right ventricular catheterization at P30. **(D)** Quantification of right ventricular systolic pressure (RVSP). Data are mean ± SD; *p < 0.05,* **p < 0.001 (one-way ANOVA with Tukey’s *post-hoc* test). n = 5 per group.

Supplementary Figure 3 Enrichment analysis of upregulated differentially expressed genes (DEGs) highlights predominant immune pathway activation. **(A)** Top 30 enriched Gene Ontology (GO) terms specific to upregulated DEGs. Terms related to immune and defense responses are prominently enriched. **(B)** Scatter plot of the top enriched GO terms, with color representing the statistical significance (-log10(padj)) and dot size indicating the number of genes in each term. **(C)** Top 20 enriched Kyoto Encyclopedia of Genes and Genomes (KEGG) pathways for upregulated DEGs. Immune-related pathways, including viral infection and cytokine-cytokine receptor interaction, dominate the list. **(D)** Bubble plot of the top enriched KEGG pathways, where bubble size represents the gene count and color indicates the enrichment significance. These analyses, focused exclusively on upregulated genes, reinforce the conclusion that immune system activation is a central transcriptional response to neonatal increased pulmonary flow (IPF).

Supplementary Figure 4 IFN-receptor blocker MAR1-5A3 attenuates IPF-induced vascular remodeling. **(A)** Representative immunofluorescence images of lung sections (α-SMA in red). **(B)** corresponding quantification of α-SMA fluorescence density from Sham, IPF, and IPF+MAR1-5A3 groups. **(C)** Representative H&E staining of lung sections. Data are mean ± SD; *p < 0.05,* **p < 0.001 (one-way ANOVA with Tukey’s *post-hoc* test). n = 5 per group.
