# Supplementary material for: Neonatal pulmonary vascular remodeling induced by increased blood flow is associated with an antiviral-like immune signature
Source: Front Immunol. 2026 Mar 4;17:1780303. doi: 10.3389/fimmu.2026.1780303 (PMC12995742; doi:10.3389/fimmu.2026.1780303)
Supplement: Supplementary file 2 [file Table1.docx]

Supplemental Table S1 Animal usage, baseline characteristics, sex distribution, and perioperative survival

Perioperative survival and baseline characteristics of neonatal IPF model

| Variable | Sham (n=30) | IPF (n=30) | *P* value |
| --- | --- | --- | --- |
| Total animals operated | 30 | 30 | / |
| Female, n (%) | 15 (50%) | 15 (50) | 1.000 |
| Age of surgery | P7 | P7 | / |
| Surgery-related mortality (0-24h), n (%) | 2 (6.7%) | 4 (13.3%) | 0.671 |
| Early mortality (24-72h), n (%) | 1 (3.3%) | 3 (10.0%) | 0.612 |
| Late mortality (24-72h), n (%) | 0 (0%) | 1 (3.3%) | 1.000 |
| Survivors at P30, n (%) | 27 (90.0%) | 22 (73.3%) | 0.181 |

CsA intervention cohort (independent cohort with within-batch matched IPF controls)

| Arm | Cohort type | Enrolled (n) | Female/Male (n/n) | Randomized within batch | Final analyzed (n) |
| --- | --- | --- | --- | --- | --- |
| IPF+Vehicle | Independent intervention cohort | 10 | 5/5 | Yes | 5 |
| IPF + CsA | Independent intervention cohort | 10 | 5/5 | Yes | 5 |
