# Supplementary material for: Neonatal pulmonary vascular remodeling induced by increased blood flow is associated with an antiviral-like immune signature
Source: Front Immunol. 2026 Mar 4;17:1780303. doi: 10.3389/fimmu.2026.1780303 (PMC12995742; doi:10.3389/fimmu.2026.1780303)
Supplement: Supplementary file 3 [file Table2.docx]

Supplemental Table S2 Primers

Primers

| Gene |  | Sequence (5'->3') |
| --- | --- | --- |
| Gbp4 | Forward | GGAGAAGCTAACGAAGGAACAA |
|  | Reverse | TTCCACAAGGGAATCACCATTTT |
| Rtp4 | Forward | TGGGAGCAGACATTTCAAGAAC |
|  | Reverse | ACCTGAGCAGAGGTCCAACTT |
| Isg15 | Forward | GGTGTCCGTGACTAACTCCAT |
|  | Reverse | TGGAAAGGGTAAGACCGTCCT |
| Spon2 | Forward | ATGGAAAACGTGAGTCTTGCC |
|  | Reverse | TGATGCTGTATCTAGCCAGAGG |
| Oas2 | Forward | TTGAAGAGGAATACATGCGGAAG |
|  | Reverse | GGGTCTGCATTACTGGCACTT |
| Mx1 | Forward | GACCATAGGGGTCTTGACCAA |
|  | Reverse | AGACTTGCTCTTTCTGAAAAGCC |
| Oas1a | Forward | GCCTGATCCCAGAATCTATGC |
|  | Reverse | GAGCAACTCTAGGGCGTACTG |
| Ifi27l2a | Forward | GCTTGTTGGGAACCCTGTTTG |
|  | Reverse | GGATGGCATTTGTTGATGTGGAG |
| Treml4 | Forward | CTGGAGGTACTCACAACTGCT |
|  | Reverse | GGCTCTGTCCTACCATTCTATGA |
| Spp1 | Forward | AGCAAGAAACTCTTCCAAGCAA |
|  | Reverse | GTGAGATTCGTCAGATTCATCCG |
| Myh11 | Forward | AAGCTGCGGCTAGAGGTCA |
|  | Reverse | CCCTCCCTTTGATGGCTGAG |
| Mmp9 | Forward | CTGGACAGCCAGACACTAAAG |
|  | Reverse | CTCGCGGCAAGTCTTCAGAG |
| Tgfb1 | Forward | CTCCCGTGGCTTCTAGTGC |
|  | Reverse | GCCTTAGTTTGGACAGGATCTG |
| Tgfb3 | Forward | CCTGGCCCTGCTGAACTTG |
|  | Reverse | TTGATGTGGCCGAAGTCCAAC |
| Tgfb2 | Forward | CTTCGACGTGACAGACGCT |
|  | Reverse | GCAGGGGCAGTGTAAACTTATT |
| Col8a1 | Forward | ACTCTGTCAGACTCATTCAGGC |
|  | Reverse | CAAAGGCATGTGAGGGACTTG |
| Loxl1 | Forward | GAGTGCTATTGCGCTTCCC |
|  | Reverse | GGTTGCCGAAGTCACAGGT |
| Smad3 | Forward | CACGCAGAACGTGAACACC |
|  | Reverse | GGCAGTAGATAACGTGAGGGA |
| Yap1 | Forward | ACCCTCGTTTTGCCATGAAC |
|  | Reverse | TGTGCTGGGATTGATATTCCGTA |
