# Supplementary figures and images for: Neonatal pulmonary vascular remodeling induced by increased blood flow is associated with an antiviral-like immune signature
Source: Front Immunol. 2026 Mar 4;17:1780303. doi: 10.3389/fimmu.2026.1780303 (PMC12995742; doi:10.3389/fimmu.2026.1780303)

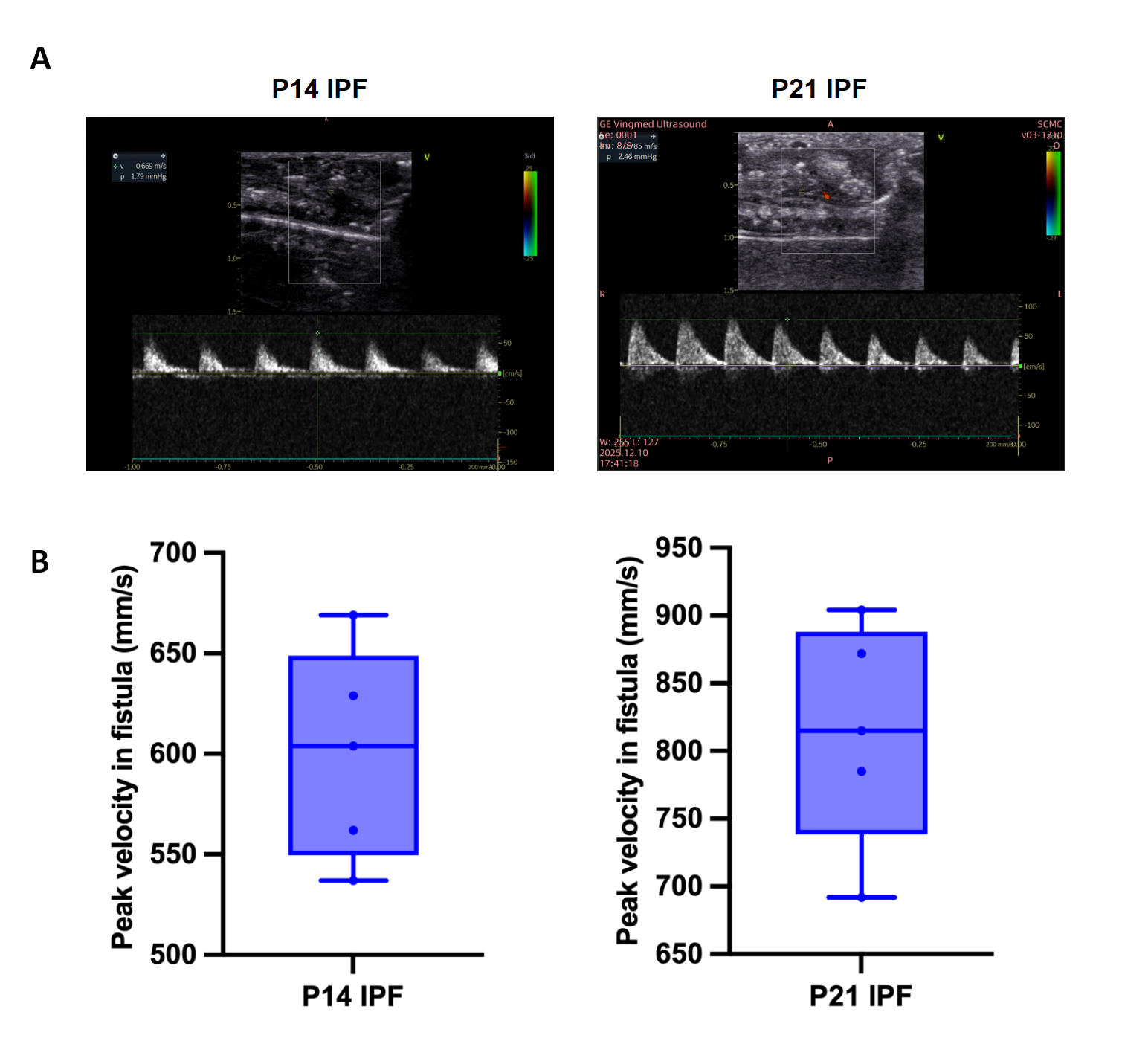

Supplement: Supplementary Figure 1 — Shunt patency and stability revealed by abdominal ultrasound imaging. (A) Representative of abdominal ultrasound imaging at P14 and P21. (B) Quantification of peak velocity at the fistula. [file Image1.tif]

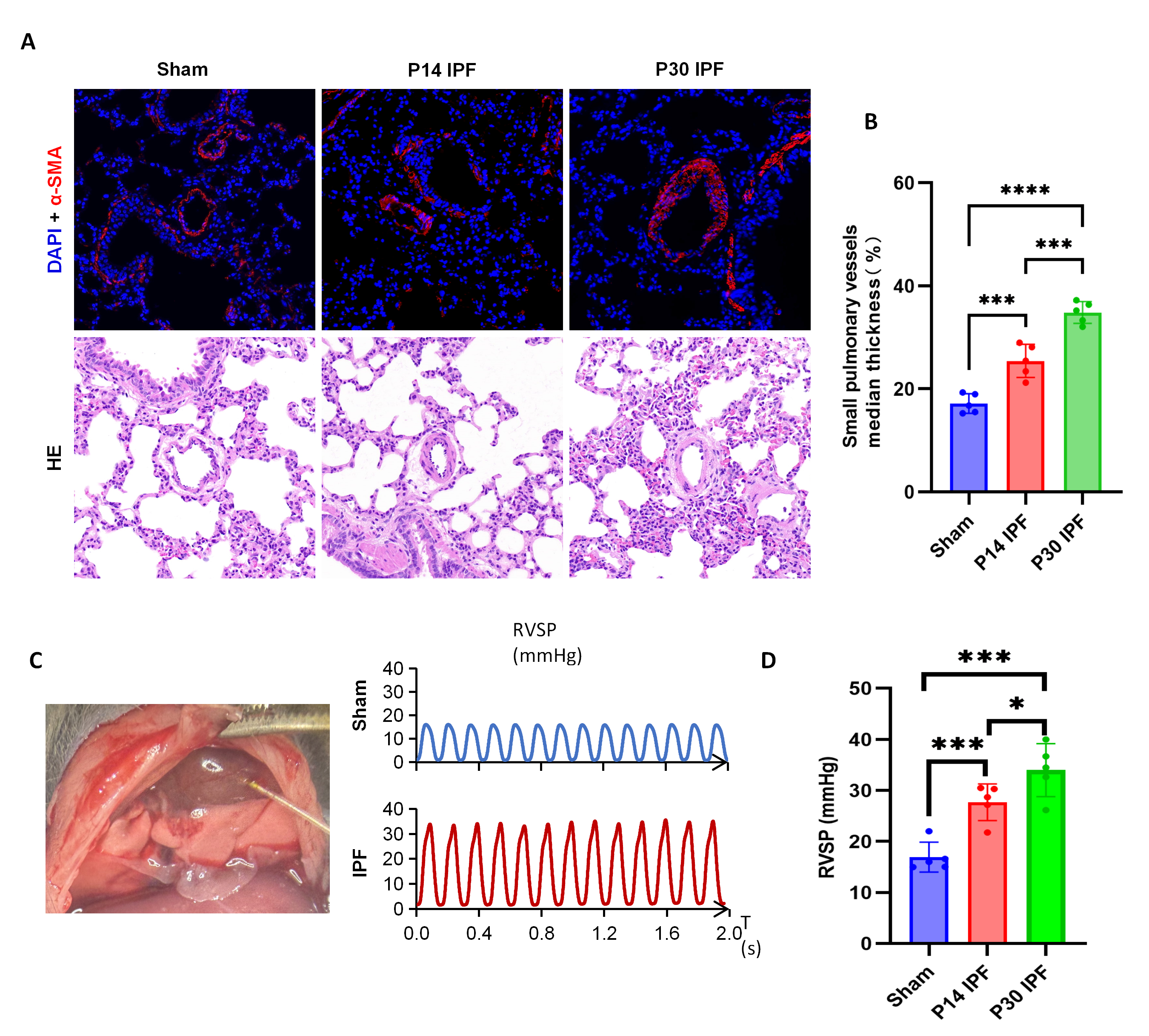

Supplement: Supplementary Figure 2 — Time-course analyses of pulmonary vessels remodeling and pulmonary vascular resistance. (A) Representative of pulmonary vessels imaging at P14 and P30. (B) Quantification of small pulmonary vessels median thickness(%). (C) Representative of right ventricular catheterization at P30. (D) Quantification of right ventricular systolic pressure (RVSP). Data are mean ± SD; *p < 0.05,* **p < 0.001 (one-way ANOVA with Tukey’s post-hoc test). n = 5 per group. [file Image2.tif]

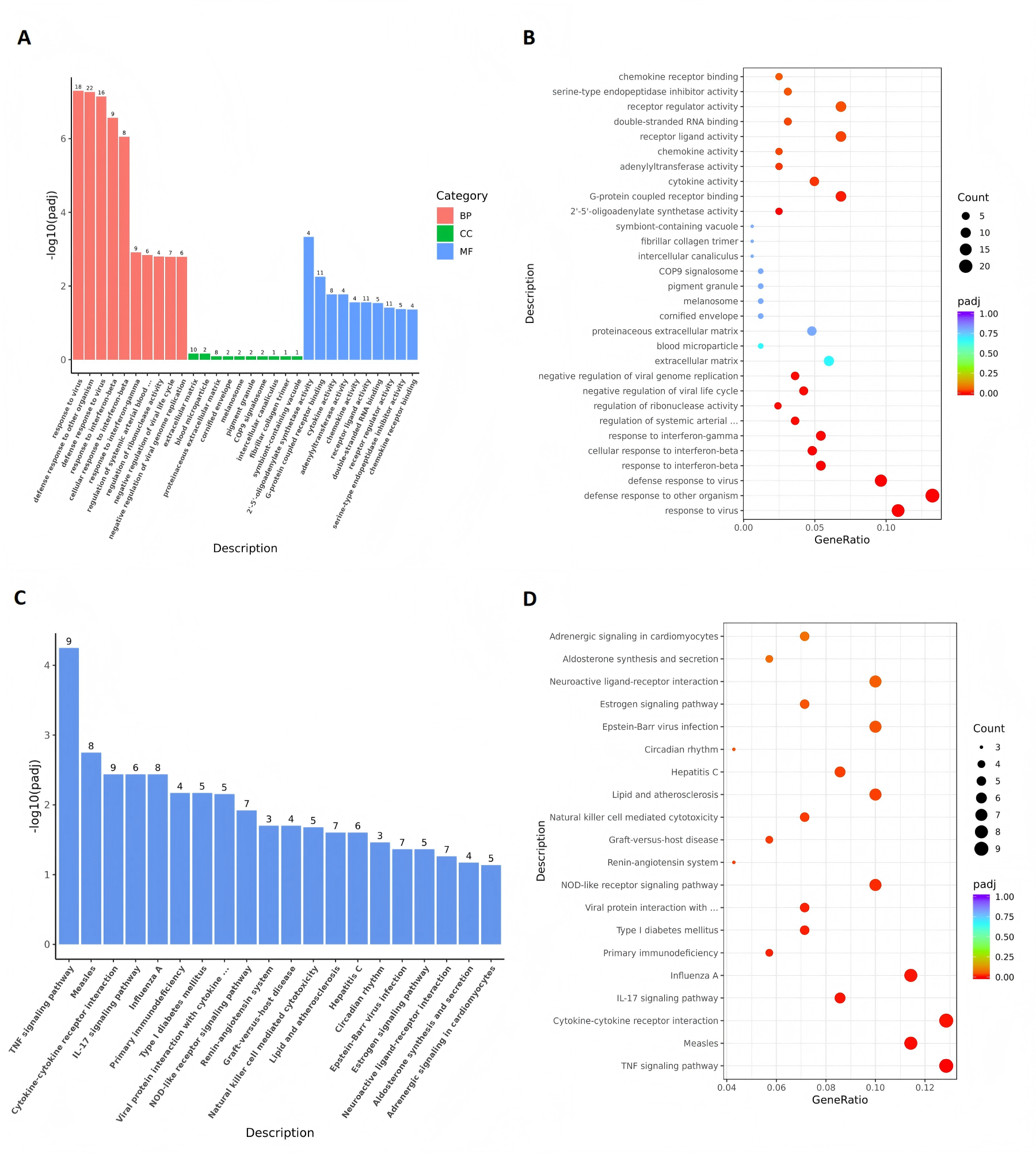

Supplement: Supplementary Figure 3 — Enrichment analysis of upregulated differentially expressed genes (DEGs) highlights predominant immune pathway activation. (A) Top 30 enriched Gene Ontology (GO) terms specific to upregulated DEGs. Terms related to immune and defense responses are prominently enriched. (B) Scatter plot of the top enriched GO terms, with color representing the statistical significance (-log10(padj)) and dot size indicating the number of genes in each term. (C) Top 20 enriched Kyoto Encyclopedia of Genes and Genomes (KEGG) pathways for upregulated DEGs. Immune-related pathways, including viral infection and cytokine-cytokine receptor interaction, dominate the list. (D) Bubble plot of the top enriched KEGG pathways, where bubble size represents the gene count and color indicates the enrichment significance. These analyses, focused exclusively on upregulated genes, reinforce the conclusion that immune system activation is a central transcriptional response to neonatal increased pulmonary flow (IPF). [file Image3.tif]

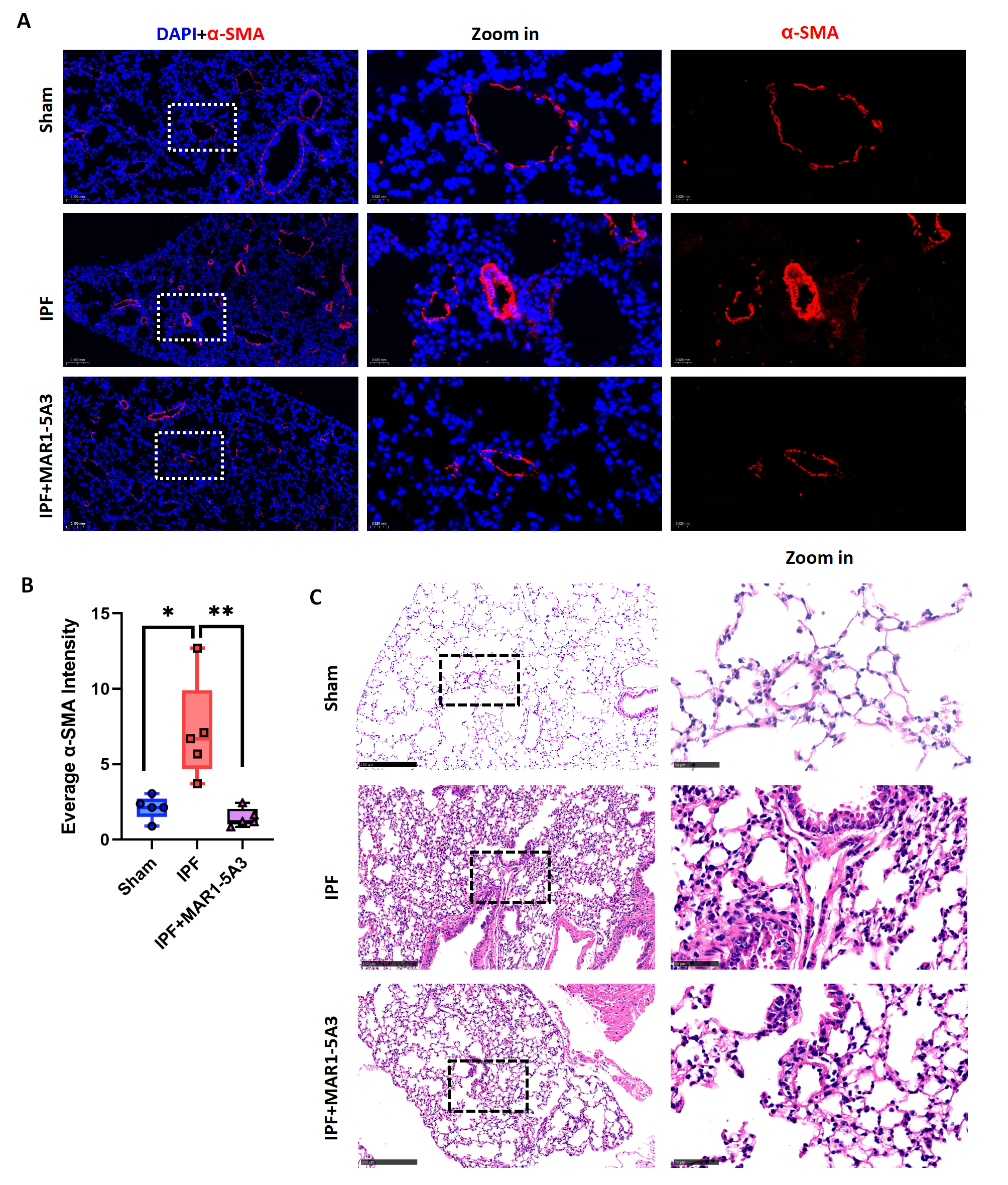

Supplement: Supplementary Figure 4 — IFN-receptor blocker MAR1-5A3 attenuates IPF-induced vascular remodeling. (A) Representative immunofluorescence images of lung sections (α-SMA in red). (B) corresponding quantification of α-SMA fluorescence density from Sham, IPF, and IPF+MAR1-5A3 groups. (C) Representative H&E staining of lung sections. Data are mean ± SD; *p < 0.05,* **p < 0.001 (one-way ANOVA with Tukey’s post-hoc test). n = 5 per group. [file Image4.tif]
